# Effects of all‐out sprint interval training under hyperoxia on exercise performance

**DOI:** 10.14814/phy2.14194

**Published:** 2019-07-29

**Authors:** Michihiro Kon, Kohei Nakagaki, Yoshiko Ebi

**Affiliations:** ^1^ School of International Liberal Studies Chukyo University Nagoya Japan; ^2^ Department of Sports Sciences Japan Institute of Sports Sciences Tokyo Japan; ^3^ Department of Sports Sciences Yamanashi Gakuin University Yamanashi Japan

**Keywords:** Accumulated oxygen deficit, hyperoxic training, lactate curve, trained athletes

## Abstract

All‐out sprint interval training (SIT) is speculated to be an effective and time‐efficient training regimen to improve the performance of aerobic and anaerobic exercises. SIT under hypoxia causes greater improvements in anaerobic exercise performance compared with that under normoxia. The change in oxygen concentration may affect SIT‐induced performance adaptations. In this study, we aimed to investigate the effects of all‐out SIT under hyperoxia on the performance of aerobic and anaerobic exercises. Eighteen college male athletes were randomly assigned to either the normoxic sprint interval training (NST, *n* = 9) or hyperoxic (60% oxygen) sprint interval training (HST, *n* = 9) group and performed 3‐week SIT (six sessions) consisting of four to six 30‐sec all‐out cycling sessions with 4‐min passive rest. They performed maximal graded exercise, submaximal exercise, 90‐sec maximal exercise, and acute SIT tests on a cycle ergometer before and after the 3‐week intervention to evaluate the performance of aerobic and anaerobic exercises. Maximal oxygen uptake significantly improved in both groups. However, blood lactate curve during submaximal exercise test significantly improved only in the HST group. The accumulated oxygen deficit (AOD) during 90‐sec maximal exercise test significantly increased only in the NST group. The average values of mean power outputs over four bouts during the acute SIT test significantly improved only in the NST group. These findings suggest that all‐out SIT might induce greater improvement in aerobic exercise performance (blood lactate curve) but impair SIT‐induced enhancements in anaerobic exercise performance (AOD and mean power output).

## Introduction

All‐out sprint interval training (SIT) has gained attention as an exercise training regimen that enhances the performance of aerobic and anaerobic exercises despite lower training volume. Burgomaster et al. (2005) demonstrated that SIT consisting of four to seven 30‐sec all‐out cycling with 4‐min recovery induces improvements in endurance time to fatigue during submaximal cycling, maximal oxygen uptake (${\dot{\text{V}}\text{O}}_{2}\max$), and power output during repeated sprint test. In addition, similar enhancements in 750 kJ cycling time (Gibala et al. 2006) and ${\dot{\text{V}}\text{O}}_{2}\max$ (Burgomaster et al. 2008; Cocks et al. 2013; Shepherd et al. 2013) are induced following SIT and traditional endurance training although total training volume is lower for SIT versus endurance training. These results suggest that all‐out SIT may be a more time‐efficient and effective training method to improve the performance of aerobic and anaerobic exercises.

In recent years, all‐out SIT under hypoxic condition has been reported to induce greater improvements in exercise performance compared with that under normoxic condition. Recent studies have shown greater improvement in power output during repeated sprint test following SIT under hypoxia compared with that under normoxia in male sprinters (Kasai et al. 2017) and female lacrosse athletes (Kasai et al. 2015). In addition, SIT under hypoxia leads to greater increases in power output and number of sprint sets until exhaustion during repeated sprint tests in male cyclists (Faiss et al. 2013) and male and female cross‐country skiers (Faiss et al. 2015). However, one study found no additional effect of hypoxic SIT on the degree of improvement in these performance parameters in endurance‐trained male subjects (Montero and Lundby 2017). Based on these results, all‐out SIT under hypoxia, when compared with that under normoxia, may be more useful for enhancing anaerobic exercise performance. Hypoxic exposure increases the contribution of the anaerobic energy system during all‐out sprint exercise (Ogura et al. 2006). Therefore, researchers speculate that the increased stimulus to the anaerobic energy system may contribute to greater improvements in anaerobic exercise performance (Kasai et al. 2015). If the alteration in the energy system contributions during SIT due to the change in oxygen concentration affects the SIT‐induced performance adaptations, all‐out SIT under hyperoxia may cause greater SIT‐induced improvement in aerobic exercise performance because hyperoxia exposure increases the percentage of energy supplied from the aerobic system during maximal exercise (Linossier et al. 2000). By contrast, SIT under hyperoxia may impair SIT‐induced enhancement in anaerobic exercise performance because hyperoxia exposure decreases the percentage of energy supplied from the anaerobic system during maximal exercise (Linossier et al. 2000).

In this study, we aimed to investigate the effects of all‐out SIT under hyperoxia on the performance of aerobic and anaerobic exercises in trained athletes. We hypothesized that all‐out SIT under hyperoxia would lead to greater enhancement in aerobic exercise performance but diminishes anaerobic exercise performance.

## Materials and Methods

### Subjects

Eighteen healthy college male athletes participated in this study. None of the subjects were smokers or taking any medications. They belonged to the canoe club at the same university and performed canoe‐specific training 5 days per week. The subjects were randomly assigned to either the normoxic sprint training (NST, *n* = 9) or the hyperoxic sprint training (HST, *n* = 9) group. The physical characteristics of the subjects are shown in Table 1. The subjects were informed of the experimental procedures, as well as the purpose of the present study. Informed consent was subsequently obtained from all subjects. The Japan Institute of Sports Sciences Ethics Committee approved the study design (Approval no. 001).

**Table 1:** Characteristics of the subjects

	NST (*n* = 9)		HST (*n* = 9)	
	Pre	Post	Pre	Post
Age (year)	20.7 ± 0.9		19 ± 0.4	
Height (cm)	172.9 ± 1.8		171.9 ± 1.8	
Body mass (kg)	72.8 ± 3.1	72.7 ± 3.2	70.6 ± 1.6	70.8 ± 1.6
Body mass index (kg/m^2^)	24.3 ± 0.8	24.3 ± 0.9	23.9 ± 0.3	24.0 ± 0.3

Values are presented as means ± SE. NST, normoxic sprint training; HST hyperoxic sprint training.

### Training protocol

This study was conducted using a single‐blind design. The subjects performed 3‐week all‐out SIT on nonconsecutive days (every Monday and Thursday or every Tuesday and Friday, six sessions in total). Before the training, the subjects in the NST and HST groups wore a face mask covering the nose and mouth. In the HST group, the subjects received hyperoxic gas (60% oxygen) from the mask via a hyperoxic generator (TOK‐20DX‐M; IBS Co., Ltd., Osaka, Japan). The subjects in the NST group received normoxic air from the mask. The HST group was exposed to hyperoxic condition from 10 min before the sprint interval exercise session until immediately after the exercise session. The SIT consisted of repeated 30‐s all‐out cycling bouts on a cycle ergometer (Excalibur Sport 925900; Lode BV, Groningen, The Netherlands) at resistance equivalent to 7.5% of their body mass with 4‐min passive rest between bouts. The cycle ergometer was set to fixed torque mode. The number of cycling bouts performed during each training session increased from four during week 1, to five during week 2, and finally to six during week 3. The subjects were instructed to sprint as fast as possible against the resistance and were encouraged verbally.

### Maximal graded exercise test

A maximal graded exercise test on the cycle ergometer was performed to determine ${\dot{\text{V}}\text{O}}_{2}\max$ and maximal workload. After a 5‐min warm‐up at 100 W, the power output was increased by 15 W every 30 sec until exhaustion. Respiratory gas samples were collected in Douglas bags every 30 sec during the test. The highest 30‐sec ${\dot{\text{V}}\text{O}}_{2}$ was regarded as ${\dot{\text{V}}\text{O}}_{2}\max$ of the test. The subjects were instructed to maintain a cadence of 90 rpm during the test. The test was terminated when they could not maintain pedal frequency within 5 rpm of the required level for 5 sec despite vigorous encouragement. The highest workload maintained for 30 sec was defined as maximal workload.

### Submaximal intermittent incremental exercise test

A submaximal intermittent incremental exercise test on the cycle ergometer was performed to determine both the blood lactate curve and ${\dot{\text{V}}\text{O}}_{2}$‐power output linear relationship. The power output for each 6‐min stage was calculated as a percentage of the maximal workload (first stage, 20%; second stage, 30%; third stage, 40%; fourth stage, 50%; and fifth stage, 60%). The subjects were instructed to maintain a cadence of 90 rpm during the test. A 2‐min rest period between each 6‐min stage was allowed for the sampling of capillary blood. The ${\dot{\text{V}}\text{O}}_{2}$ values for the last 2 min of each 6‐min stage were recorded and used to determine the ${\dot{\text{V}}\text{O}}_{2}$‐power output linear relationship for each subject.

### 90‐sec maximal exercise test

The subjects performed a 90‐sec maximal exercise test on the cycle ergometer as described previously (Gastin and Lawson 1994). Before the trial, subjects were given a 5‐min warm‐up at 100 W and then a 3‐min rest. The resistance was reduced from 9.5 to 7.5% of their body weight at 30 sec and further reduced to 5.5% of their body weight at 60 sec. For an all‐out effort, the subjects were instructed and strongly encouraged to maintain the cadence as high as possible throughout the test. The estimated oxygen demand for the 90‐sec maximal exercise test was then calculated by extrapolation from the ${\dot{\text{V}}\text{O}}_{2}$‐power output linear relationship. The accumulated oxygen deficit (AOD) was calculated as the difference between the estimated oxygen demand of exercise and the accumulated oxygen uptake (AOU). Average power output, estimated oxygen demand and oxygen uptake were calculated over 30‐sec intervals.

### Sprint interval exercise test

The subjects performed an all‐out sprint interval exercise test under normoxic conditions, comprising four 30‐sec maximal cycling bouts with 4‐min passive rest between bouts using the cycle ergometer. The resistance was equivalent to 7.5% of their body weight. The peak and mean power output values of each bout were measured and recorded.

### Cardiorespiratory measurements

${\dot{\text{V}}\text{O}}_{2}$ and ${\dot{\text{V}}\text{CO}}_{2}$ were determined using the Douglas bag method. The O_2_ and CO_2_ fractions in the expired gas were measured with a calibrated gas analyzer (Aeromonitor AE310s; Minato Medical Science, Osaka, Japan). The expired gas volume was determined using a dry gas meter (Oval GAL‐55; Oval Corp., Tokyo, Japan).

### Blood sampling and analysis

Blood sampling was performed before (pre) and after (post) the 3‐week intervention. In the morning (between 08:00 and 09:00), all subjects visited the laboratory after overnight fasting and rested for 30 min before the blood collection. The subjects were confirmed to ensure a 48‐h period without any exercise activity prior to the blood collection. Subsequently, blood samples were collected from each subject’s forearm. Serum samples were obtained by centrifugation (3000 rpm for 15 min) and stored at −80°C until analysis. Serum derivatives of reactive oxygen metabolites (d‐ROMs), which are markers to evaluate hydroperoxide levels (oxidative stress), and serum biological antioxidant potential (BAP), which indicates antioxidant capacity, were measured using a FREE Carrio Duo (Wismerll Co., Ltd., Tokyo, Japan). The BAP/d‐ROMs ratio was also measured to evaluate serum oxidant‐antioxidant balance (Sone et al. 2019). Blood lactate concentration was determined using a Biosen S‐Line (EKF‐diagnostic GmbH, Barleben, Germany) from 20 *µ*L of fingertip capillary blood sample. Oxyhemoglobin saturation (SpO2) was measured using a forehead pulse oximeter (Masimo Rad‐57; Masimo Corp., CA).

### Nutritional and physical activity controls

During the experimental period, the subjects were ordered to continue their normal diet and physical activity. They were also instructed not to perform any exercise for 48 h before the exercise performance tests.

### Statistical analysis

Results are presented as means with standard errors (SE). All data were analyzed using a two‐way ANOVA with repeated measures. When significant differences existed, a post hoc analysis test (Fisher’s least significant difference) was performed. The percent changes between the groups were compared using unpaired *t* tests. The level of statistical significance was set at *P* < 0.05.

## Results

### Body composition

Before the 3‐week training period, no significant difference was found in the physical characteristics between the NST and HST groups (Table 1). Body mass (main effect, *P* = 0.57) and body mass index (main effect, *P* = 0.56) did not change after the training period in either group.

### Oxyhemoglobin saturation

**Figure 1:**
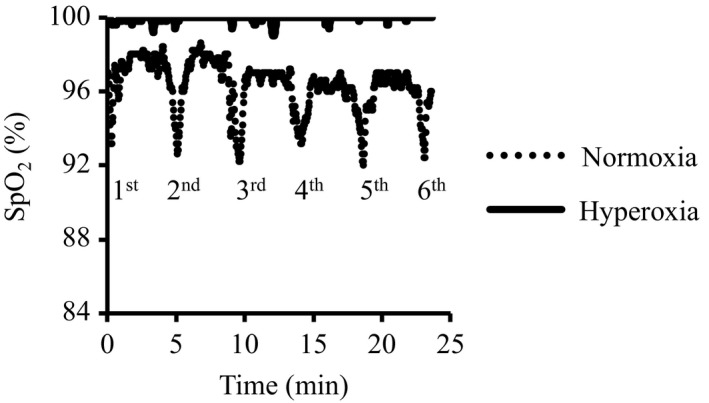
Typical example of changes in SpO_2_ during six repeated bouts of sprint exercise under normoxia and hyperoxia obtained from the same subject.

Figure 1 shows a typical example of changes in SpO_2_ during six repeated bouts of sprint exercise under normoxia and hyperoxia obtained from the same subject. The SpO_2_ during SIT under normoxia changed between 98% and 92%. By contrast, no change (almost 100%) in SpO_2_ was observed during SIT under hyperoxia.

### Training power output

**Figure 2:**
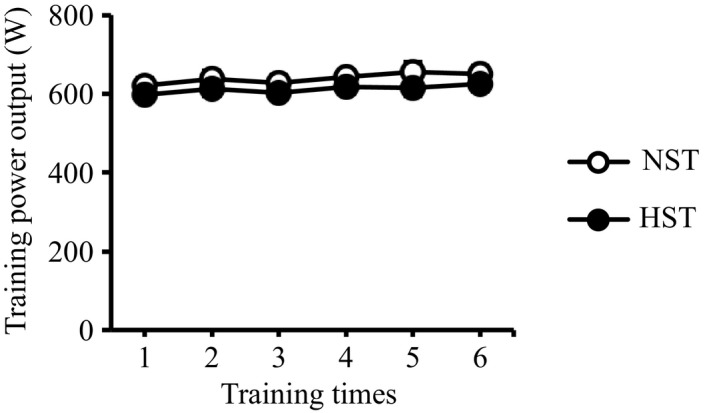
Training power outputs throughout the 3‐week all‐out sprint interval training. NST, normoxic sprint interval training (*n* = 9); HST, hyperoxic sprint interval training (*n* = 9). Values are presented as means ± SE.

Figure 2 shows the average values of mean power outputs over four, five, or six bouts throughout the 3‐week training period. No significant differences were found in the power outputs throughout the 3‐week training period between the NST and HST groups (main effect, *P* = 0.31).

### ${\dot{\text{V}}\text{O}}_{2}\max$and maximal workload during maximal graded exercise test

**Figure 3:**
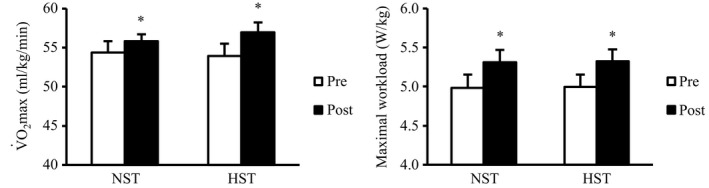
Maximal oxygen uptake (${\dot{\text{V}}\text{O}}_{2}\max$) and workload before (pre) and after (post) 3 weeks of all‐out sprint interval training. NST, normoxic sprint interval training (*n* = 9); HST, hyperoxic sprint interval training (*n* = 9). Values are presented as means ± SE. *P < 0.05 versus pre.

${\dot{\text{V}}\text{O}}_{2}\max$ significantly improved in both the NST and HST groups (main effect, *P* < 0.05; Fig. 3). However, no significant difference was found in the degree of improvement in ${\dot{\text{V}}\text{O}}_{2}\max$ between the NST (3.0 ± 2.1%) and HST (6.0 ± 1.8%) groups (main effect, *P* = 0.83). Maximal workload during progressive exercise test to determine ${\dot{\text{V}}\text{O}}_{2}\max$ also significantly increased in both the NST and HST groups (*P* < 0.05; Fig. 3), with no difference between the two groups (main effect, *P* = 0.97).

### Respiratory gas and blood lactate during submaximal exercise test

Table 2 shows changes in ${\dot{\text{V}}\text{O}}_{2}$, ${\dot{\text{V}}\text{CO}}_{2}$, and respiratory exchange ratio (RER) during submaximal intermittent incremental exercise test. The ${\dot{\text{V}}\text{O}}_{2}$, ${\dot{\text{V}}\text{CO}}_{2}$, and RER progressively increased during the submaximal intermittent incremental test in the NST and HST groups (main effect, *P* < 0.05). Significant differences were not observed in ${\dot{\text{V}}\text{O}}_{2}$ (NST, main effect, *P* = 0.79; HST, main effect, *P* = 0.56) and ${\dot{\text{V}}\text{CO}}_{2}$ (NST, main effect, *P* = 0.87; HST, main effect, *P* = 0.71) between before (pre) and after (post) training in normoxia and hyperoxia. By contrast, the RER at the first stage in the NST group significantly increased after the training (*P* < 0.05, effect size = 0.78).

**Table 2:** Changes in ${\dot{\text{V}}\text{O}}_{2}$, ${\dot{\text{V}}\text{CO}}_{2}$, and RER during submaximal intermittent incremental exercise test

		NST (*n* = 9)			HST (*n* = 9)	
		Pre	Post		Pre	Post
${\dot{\text{V}}\text{O}}_{2}$ (mL/kg/min)	1st	19.2 ± 0.4	19.4 ± 0.3	1st	19.4 ± 0.4	19.1 ± 0.5
	2nd	23.9 ± 0.6	23.7 ± 0.4	2nd	24.3 ± 0.5	23.8 ± 0.6
	3rd	29.2 ± 0.8	29.1 ± 0.5	3rd	29.4 ± 0.7	28.8 ± 0.9
	4th	34.6 ± 0.9	34.3 ± 0.6	4th	35.0 ± 0.9	34.2 ± 1.1
	5th	40.7 ± 1.2	39.9 ± 0.7	5th	41.0 ± 1.0	40.0 ± 1.2
${\dot{\text{V}}\text{CO}}_{2}$ (mL/kg/min)	1st	17.2 ± 0.4	17.9 ± 0.4	1st	17.3 ± 0.6	17.4 ± 0.5
	2nd	21.5 ± 0.5	22.0 ± 0.5	2nd	22.3 ± 0.6	22.3 ± 0.5
	3rd	27.1 ± 0.8	27.2 ± 0.5	3rd	27.8 ± 0.8	27.4 ± 0.9
	4th	33.0 ± 0.8	32.8 ± 0.7	4th	34.3 ± 1.2	33.4 ± 1.2
	5th	40.4 ± 1.3	40.0 ± 0.9	5th	41.6 ± 1.4	40.6 ± 1.2
RER	1st	0.89 ± 0.01	0.93 ± 0.02*	1st	0.89 ± 0.02	0.91 ± 0.01
	2nd	0.90 ± 0.01	0.93 ± 0.02	2nd	0.92 ± 0.01	0.94 ± 0.01
	3rd	0.93 ± 0.01	0.94 ± 0.01	3rd	0.94 ± 0.01	0.95 ± 0.01
	4th	0.96 ± 0.01	0.96 ± 0.01	4th	0.98 ± 0.01	0.98 ± 0.01
	5th	0.99 ± 0.01	1.00 ± 0.01	5th	1.01 ± 0.01	1.01 ± 0.01

Values are presented as means ± SE. NST, normoxic sprint training; HST hyperoxic sprint training.

* *P* < 0.05 versus Pre.

**Figure 4:**
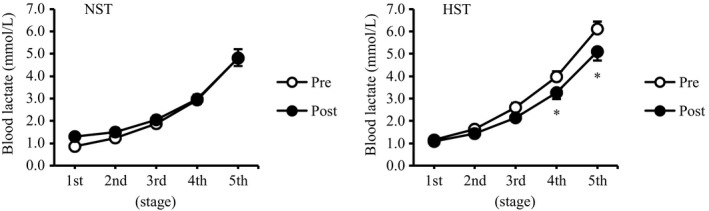
Blood lactate curve during submaximal intermittent incremental cycling test before (pre) and after (post) 3 weeks of all‐out sprint interval training. NST, normoxic sprint interval training (*n* = 9); HST, hyperoxic sprint interval training (*n* = 9). Values are presented as means ± SE. *P < 0.05 versus pre.

Figure 4 shows blood lactate data during the submaximal intermittent incremental cycling test. Blood lactate also progressively increased in the NST and HST groups (main effect, *P* < 0.05). However, blood lactate levels at the 4th (*P* < 0.05, effect size = 0.80) and 5th (*P* < 0.05, effect size = 0.86) stages after the training were significantly lower than those before the training in the HST group, but no significant differences were found in the NST group (*P* = 0.51).

### Power outputs, AOU, AOD, and %AOD during 90‐s maximal exercise test

**Figure 5:**
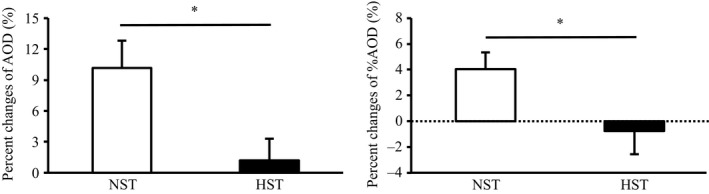
Percent changes in AOD and %AOD during 90‐sec maximal cycling test before (pre) and after (post) 3 weeks of all‐out sprint interval training. NST, normoxic sprint interval training (*n* = 9); HST, hyperoxic sprint interval training (*n* = 9). Values are presented as means ± SE. *P < 0.05 between the NST and HST.

Table 3 shows power outputs, AOU, AOD, and %AOD data during 90‐sec maximal exercise test. Peak and mean power outputs significantly increased in both the groups (main effect, *P* < 0.05). Although no significant differences were found in the degree of improvements in peak and mean power outputs between the NST and HST groups, percent changes in the peak and mean power outputs in the NST group (peak power output; 10.9 ± 3.5%, mean power output; 6.3 ± 1.7%) tended to be higher than those in the HST group (peak power output; 3.5 ± 2.3%, mean power output; 2.2 ± 1.0%) (*P* < 0.10). AOU significantly increased in both the NST and HST groups after the training (main effect, *P* < 0.05). However, AOD (*P* < 0.05, effect size = 0.85) and %AOD (*P* < 0.05, effect size = 0.61) significantly increased only in the NST group after the training. In addition, the NST group showed significantly greater percentage increases in AOD (*P* < 0.05, effect size = 1.07) and %AOD (*P* < 0.05, effect size = 0.92) than the HST group (Fig. 5).

**Table 3:** Changes in peak and mean power outputs, AOU, AOD, and %AOD during 90‐sec maximal exercise test

	NST (*n* = 9)		HST (*n* = 9)	
	Pre	Post	Pre	Post
Peak power (W/kg)	20.5 ± 0.7	22.7 ± 1.1*	20.3 ± 0.6	21.0 ± 0.7*
Mean power (W/kg)	6.9 ± 0.1	7.3 ± 0.2*	7.0 ± 0.1	7.2 ± 0.1*
AOU (mL/kg)	65.0 ± 1.2	66.2 ± 1.2*	68.1 ± 1.5	69.7 ± 1.3*
AOD (mLO_2_eq/kg)	55.4 ± 1.7	61.0 ± 2.4*	55.6 ± 1.2	56.3 ± 1.9
%AOD	46.0 ± 0.8	47.9 ± 1.2*	45.0 ± 0.9	44.6 ± 1.2

Values are presented as means ± SE. AOU, accumulated oxygen uptake; AOD, accumulated oxygen deficit; NST, normoxic sprint training; HST hyperoxic sprint training.

* *P* < 0.05 versus Pre.

### Power outputs during acute sprint interval exercise test

**Figure 6:**
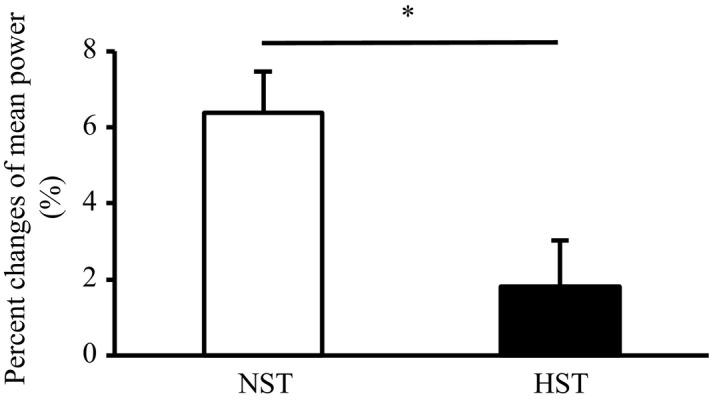
Percent changes in average mean power outputs during acute sprint interval exercise test before (pre) and after (post) 3 weeks of all‐out sprint interval training. NST, normoxic sprint interval training (*n* = 9); HST, hyperoxic sprint interval training (*n* = 9). Values are presented as means ± SE. *P < 0.05 between the NST and HST.

Table 4 shows peak and mean power output data during acute sprint interval test. In the NST and HST groups, the peak and mean power outputs gradually decreased before (pre) and after (post) the training period (main effect, *P* < 0.05). The average values of peak power outputs over four bouts significantly increased after the training in both the NST and HST groups (main effect, *P* < 0.05). By contrast, the average values of mean power outputs over four bouts significantly increased after the training only in the NST group (*P* < 0.05, effect size = 0.84). In addition, the NST group showed a significantly greater percentage increase in the average values of mean power output over four bouts than the HST group (*P* < 0.05, effect size = 1.12; Fig. 6).

**Table 4:** Changes in peak and mean power during acute sprint interval exercise test

		NST (*n* = 9)		HST (*n* = 9)	
		Pre	Post	Pre	Post
Peak power (W/kg)	Bout 1	20.0 ± 0.6	21.8 ± 0.8	19.4 ± 0.7	20.0 ± 0.5
	Bout 2	18.3 ± 0.5#	19.9 ± 0.6#	18.0 ± 0.5#	19.2 ± 0.6#
	Bout 3	16.5 ± 0.4#	17.9 ± 1.0#	15.6 ± 0.7#	17.1 ± 0.6#
	Bout 4	14.7 ± 0.5#	15.9 ± 1.2#	14.0 ± 0.7#	15.3 ± 0.9#
	Average	17.4 ± 0.4	18.9 ± 0.8*	16.7 ± 0.6	17.9 ± 0.6*
Mean power (W/kg)	Bout 1	9.8 ± 0.2	10.3 ± 0.3	9.9 ± 0.1	9.9 ± 0.1
	Bout 2	8.8 ± 0.2#	9.3 ± 0.2#	8.9 ± 0.1#	9.0 ± 0.1#
	Bout 3	7.9 ± 0.2#	8.4 ± 0.2#	7.8 ± 0.1#	8.1 ± 0.1#
	Bout 4	7.3 ± 0.3#	8.0 ± 0.3#	7.5 ± 0.2#	7.6 ± 0.2#
	Average	8.4 ± 0.2	9.0 ± 0.2*	8.5 ± 0.1	8.7 ± 0.1

Values are presented as means ± SE. NST, normoxic sprint training; HST hyperoxic sprint training.

^#^ *P* < 0.05 versus Bout 1.

* *P* < 0.05 versus Pre.

### Serum d‐ROM, BAP, and BAP/d‐ROMs ratio

**Figure 7:**
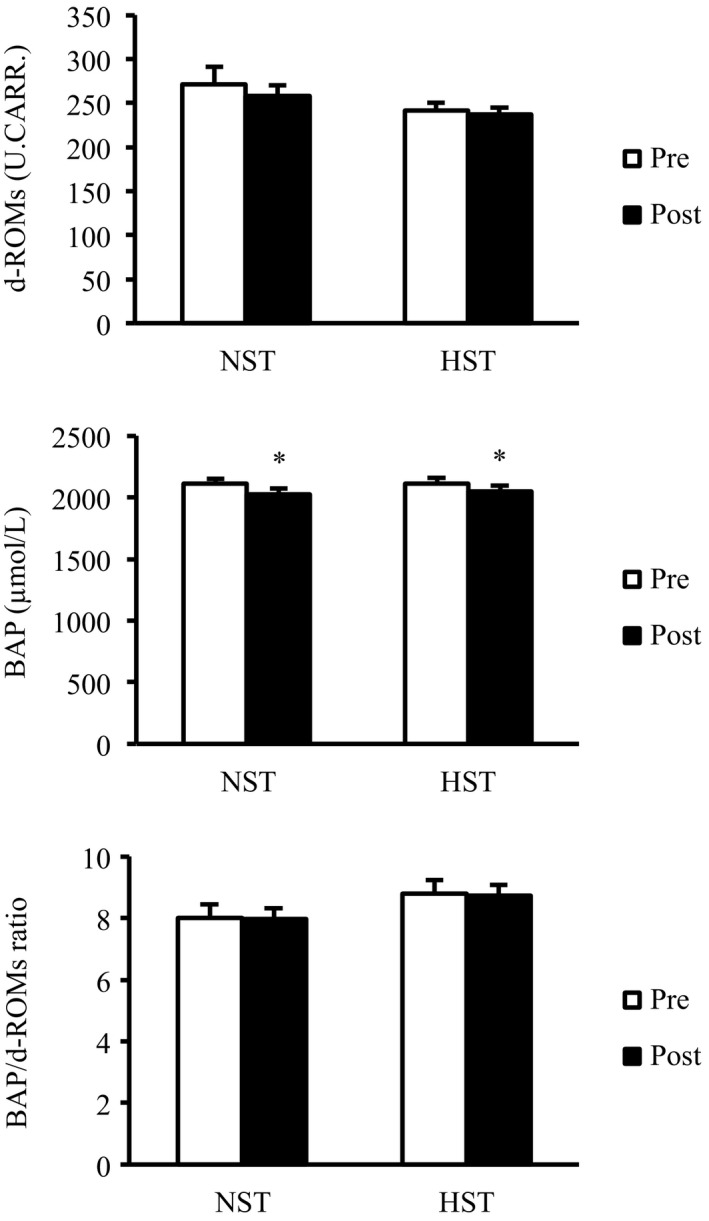
Serum derivatives of reactive oxygen metabolites (d‐ROMs), biological antioxidant potential (BAP), and BAP/d‐ROMs ratio before (pre) and after (post) 3 weeks of all‐out sprint interval training. NST, normoxic sprint interval training (*n* = 9); HST, hyperoxic sprint interval training (*n* = 9). Values are presented as means ± SE. *P < 0.05 versus pre.

Figure 7 shows serum d‐ROMs, BAP, and BAP/d‐ROMs data before (pre) and after (post) 3‐week SIT. Serum d‐ROMs did not change in either the NST or HST groups. By contrast, serum BAP significantly decreased after the training in both groups (main effect, *P* < 0.05). However, the BAP/d‐ROMs ratio did not change in either the NST or HST groups.

## Discussion

This study investigated the effects of all‐out SIT under hyperoxia on the performance of aerobic and anaerobic exercises. The blood lactate curve during the submaximal intermittent incremental cycling test, which represents enhancement in aerobic endurance capacity (Faude et al. 2009), improved only in the HST group. By contrast, hyperoxia exposure impaired the SIT‐induced enhancements in the AOD and %AOD during 90‐s maximal exercise test. Moreover, the SIT‐induced increase in average mean power output during sprint interval exercise test was also diminished by hyperoxia exposure (Table 4). These present results suggest that all‐out SIT under hyperoxia may bring about greater improvement in aerobic exercise performance (blood lactate curve) but impair SIT‐induced enhancements in anaerobic exercise performance (AOD, %AOD, and mean power output).

In the present study, the blood lactate curve during submaximal cycling improved after the training period only in the HST group, but no difference was found in the degree of improvement in ${\dot{\text{V}}\text{O}}_{2}\max$ between the NST and HST groups. Perry et al. (2005) demonstrated that hyperoxic (60% oxygen) interval training (10 repeats of 4 min cycling at 90% heart rate max with 2 min recovery, 3 days/week for 6 weeks) leads to greater enhancement in cycling performance time to exhaustion at 90% ${\dot{\text{V}}\text{O}}_{2}\max$ without greater improvement in ${\dot{\text{V}}\text{O}}_{2}\max$ when compared with normoxic interval training. These present and previous results suggest that hyperoxic interval training may induce greater improvement in aerobic exercise performance without greater enhancement of ${\dot{\text{V}}\text{O}}_{2}\max$. However, the underlying mechanism of improving aerobic exercise performance (blood lactate curve) by SIT under hyperoxia was not elucidated in this study. The improvement in the lactate curve may be related to the enhancement of the mitochondrial oxidative capacity of working muscle by exercise training. Perry et al. (2007) demonstrated that hyperoxic (60% oxygen) interval training (10 repeats of 4 min cycling at 90% ${\dot{\text{V}}\text{O}}_{2}\max$ with 2 min recovery, 3 days/week for 6 weeks) does not affect the degree of improvement in mitochondrial oxidative capacity of working muscles when compared with normoxic interval training. However, their research used submaximal interval training model, and an investigation of an all‐out SIT model has not been conducted so far. Thus, future research could investigate the effect of all‐out SIT under hyperoxia on skeletal muscle mitochondrial oxidative capacity.

All‐out SIT under hyperoxia impaired the improvements in anaerobic exercise performance, such as AOD, %AOD, and mean power output during cycling exercise tests, in this study. In a previous study, hyperoxia exposure decreases energy supply from the anaerobic system during maximal cycling exercise (Linossier et al. 2000). Therefore, in this study, hyperoxia exposure might decrease energy supply from the anaerobic system during SIT. The decreased anaerobic energy release due to hyperoxia might induce impairment in SIT‐induced improvements in anaerobic exercise performance in this study.

Additionally, the all‐out SIT‐induced enhancements in anaerobic exercise performance may be related to the increase in adenosine triphosphate production via the glycolytic system. Short‐term all‐out SIT increases glycogen content in muscles (Burgomaster et al. 2005; Gibala et al. 2006). The increased glycogen content in muscles may be induced by increases in muscle content and translocation to plasma membrane of glucose transporter 4 (GLUT4), which facilitates glucose uptake in the skeletal muscles. Hyperoxia exposure decreases GLUT4 content and/or translocation in the skeletal muscles (Bandali et al. 2003). Conversely, short‐term all‐out SIT increases GLUT4 content in the skeletal muscles (Burgomaster et al. 2007). Thus, hyperoxia exposure may diminish the SIT‐induced increase in GLUT4 and glycogen contents in skeletal muscle, which may lead to impaired SIT‐induced enhancements of anaerobic exercise performance in the HST group. Future studies are needed to clarify the effects of all‐out SIT under hyperoxia on the GLUT4 and glycogen contents in skeletal muscles.

A previous study reported that 3‐week all‐out SIT (four to six 30‐sec all‐out cycling with 4‐min recovery, 3 days/week) attenuates oxidative stress in healthy humans (Bogdanis et al. 2013). In addition, 3‐week endurance exercise training (30‐min moderate‐intensity cycling, 5 days/week) under hyperbaric hyperoxia does not increase systemic oxidative stress in young male soccer players (Burgos et al. 2016). However, to date, no study has investigated the effect of SIT under normobaric hyperoxia on oxidative stress in healthy humans. In this study, no significant difference was observed in oxidative stress (d‐ROMs) and oxidant‐antioxidant balance (BAP/d‐ROMs) between before and after SIT. To the best of our knowledge, this study is the first to determine that exercise training under normobaric hyperoxia does not affect oxidative stress in healthy humans.

This study has a several limitations. First, the present study did not use a crossover design and had a small sample size. Future studies with a crossover design and larger sample size are necessary to confirm our findings. Second, this study could not elucidate the factors that induced the performance adaptations to hyperoxic all‐out SIT because we could not investigate skeletal muscle adaptations by all‐out SIT under hyperoxia. Therefore, detailed studies using human skeletal muscles are warranted in future research. Finally, the present study did not assess nutritional status, but we instructed the subjects to continue their normal diet. Thus, future studies including assessment of nutritional status are needed.

## Conclusions

All‐out SIT under hyperoxia might induce greater improvement in aerobic exercise performance (blood lactate curve), but impairs SIT‐induced enhancements in anaerobic exercise performance (AOD, %AOD, and mean power output).

## Conflict of Interest

None declared.
